# Rosemary Extract: Phytochemical Composition and Potential for Eliminating Polymicrobial Biofilm of *Candida albicans* and Multidrug-Resistant Bacteria

**DOI:** 10.3390/biotech14030061

**Published:** 2025-08-13

**Authors:** Tuana Mendonça Faria Cintra, Raquel Teles de Menezes, Lara Steffany de Carvalho, Leticia de Miguel Nazario, Leandro Wang Hantao, Maria Cristina Marcucci, Luciane Dias de Oliveira, Vanessa Marques Meccatti-Domiciano

**Affiliations:** 1 Department of Biosciences and Oral Diagnosis, Institute of Science and Technology, São Paulo State University (ICT-UNESP), São José dos Campos 12245-000, Brazil; tuana.cintra@unesp.br (T.M.F.C.);; 2 Institute of Chemistry—University of Campinas LCGC Separations Group, Campinas 13083-862, Brazil

**Keywords:** multiresistant bacteria, microbial drug resistance, *Candida albicans*, polymicrobial biofilm, phytotherapy, herbal medicine, *Rosmarinus officinalis*

## Abstract

Herbal medicines can be promising for the treatment of infections caused by multidrug-resistant microorganisms. This study aimed to evaluate *Rosmarinus officinalis* (Rosemary) hydroalcoholic extract (RHE) regarding its phytochemical composition and potential for eliminating polymicrobial biofilm of *Candida albicans* with multidrug-resistant bacteria (*Acinetobacter baumannii*, *Klebsiella pneumoniae*, and *Pseudomonas aeruginosa*). The extraction and quantification of the extract (flavonoids and phenols) were performed, and its antioxidant activity (DPPH) and the presence of bio-active compounds were investigated using high-performance liquid chromatography with Diode Array Detection (HPLC-DAD) and Gas Chromatography–Mass Spectrometry (GC-MS). The minimum inhibitory concentration (MIC) and minimum microbicidal concentration (MMC) were determined, and the extract’s action on polymicrobial biofilms was evaluated using the MTT assay. Data were analyzed using one-way ANOVA and Tukey’s tests, as well as Kruskal–Wallis and Dunn’s tests, with a significance level of 5%. RHE showed compatible amounts of flavonoids and phenols, with an EC50 of 19.53 µg/mL. Through HPLC-DAD and GC-MS, biomolecules such as rosmarinic acid and α-Pinene were identified. The extract exhibited microbicidal activity and antibiofilm action, with reduction percentages of up to 69.6% (*p* < 0.05), showing superior performance compared to 0.12% chlorhexidine against *C. albicans* + *A. baumannii*. In conclusion, RHE may be a promising therapeutic agent against multidrug-resistant pathogens.

## 1. Introduction

In recent years, there has been an increase in cases of death from multidrug-resistant bacteria [1]. This bacterial resistance can be caused by the indiscriminate use of drugs and by genetic mutations [2]. This resistance makes conventional medicines ineffective, thus posing a threat to human health [3].

In 2024, The World Health Organization (WHO) expanded the list of microorganisms that present a major threat to society. *Pseudomonas aeruginosa*, *Acinetobacter baumannii*, and *Klebisiella pneumoniae* are still considered priorities on the updated list [4]. These microorganisms are associated with severe hospital admissions and high mortality rates, underscoring the critical need for effective control methods against these pathogens, which are implicated in the development of pneumonia, bacteremia, meningitis, and urinary tract infections [5].

These bacteria have a high capacity to spread in hospital environments and develop resistance to a wide variety of antibiotics due to several mechanisms, including the reduced permeability of the outer membrane of cells, enzyme inactivation, efflux pump systems, and the ability to form biofilms [6,7]. Bacterial biofilms are a survival strategy that bacteria use to resist the effects of antibiotics. Reports in the literature indicate that the types of species present in the biofilm and the specific antibiotics used can significantly influence the effectiveness of the treatment in disrupting the biofilm [8].

When discussing biofilms associated with nosocomial infections, the fungus *Candida albicans* is directly related, as it is commonly found in large quantities on urinary catheters and endotracheal tubes used in hospitalized patients [9,10]. Effective antifungal therapy is crucial for successful treatment. However, cases of antifungal resistance are becoming increasingly frequent, particularly with *Candida albicans* [11,12]. This fungus species is also found in the oral cavity and is the primary cause of oral candidiasis. Its ability to adhere to epithelial cells and its potential to transform into hyphal forms make it prevalent in polymicrobial biofilms [13,14].

Polymicrobial infections resulting from the interaction of bacteria, fungi, and other parasites cause serious health conditions that are difficult to manage, presenting a significant challenge to the scientific community [15].

One of the greatest advances in medicine was the creation of antibiotics to treat microbial infections, but the resistance of these bacteria has become a threat to human health [16]. To combat multi-resistant bacteria, natural products are a promising strategy against these pathogenic microorganisms. In this context, the use of rosemary-based extracts, for instance, can slow down antimicrobial resistance and act as a potential pharmacotherapeutic agent in combating these bacteria [17].

*Rosmarinus officinalis* L. or *Salvia rosmarinus* Spenn, popularly called rosemary, is an aromatic shrub native to the Mediterranean Basin [18]. Rosemary is traditionally used as tea and as a seasoning for food, but it is also utilized in the cosmetic and pharmaceutical industries [19,20]. The antimicrobial action of rosemary has been widely reported in the literature against various types of bacteria and fungi, such as *Escherichia coli*, *Klebsiella pneumoniae*, *Streptococcus mutans*, and *Pseudomonas aeruginosa* [21], *Candida albicans*, *C. dubliniensis*, *C. glabrata*, *C. krusei*, and *C. tropicalis* [13]. This antimicrobial action is associated with the presence of phytocompounds such as caffeic acid, carnosic acid, chlorogenic acid, oleanolic acid, rosmarinic acid, ursolic acid, alpha-pinene, camphor, carnosol, eucalyptol, rosmadial, and rosmanol [22,23].

Another method that has been studied is the combination of rosemary essential oil with conventional antibiotics. This combination has demonstrated synergistic results against strains of multidrug-resistant bacteria, offering a potential way to combat antimicrobial resistance [21]. In addition to its antimicrobial and antifungal properties, rosemary also exhibits several other biological benefits, including antioxidant activity, hepatoprotective effects, and even insecticidal uses [24]. One particularly noteworthy property of rosemary is its neuroprotective action, which can combat oxidative and neurotoxic damage. This effect is attributed to the presence of rosmarinic acid in its composition [25].

Given the therapeutic properties of rosemary extract reported in the literature, it is very important to verify its potential effectiveness against polymicrobial biofilms formed by *P. aeruginosa*, *A. baumannii*, *K. pneumoniae,* and *C. albicans*, which could provide a possible alternative to conventional biofilm control methods. Therefore, the aim of this study was to evaluate the rosemary extract regarding its phytochemical composition, antioxidant activity, and antibacterial effects against polymicrobial biofilms associating multidrug-resistant bacterial strains with *C. albicans*. The null hypothesis asserts that the extract does not possess antioxidant, antibacterial, or anti-biofilm properties.

## 2. Materials and Methods

### 2.1. Preparation and Phytochemical Analysis of the Extract

The hydroalcoholic extract of rosemary was prepared from branches dried at 20–27 °C for 5 days and powdered in a blender. The chosen extraction vehicle was a combination of absolute ethanol (99.5% ethyl alcohol—Merck Darmstadt, Darmstadt, Germany) and ultrapure water obtained from a Milli-Q^®^ system (EtOH:H_2_O/50:50), following the ratio of 30 g of raw material for every 100 mL of the vehicle, with an extraction time of 48 h under stirring (70 rpm, COLEMAN, TS-200 = VDRL Shaker, São Paulo, Brazil). Filtration was then carried out in two stages: first, to remove solid residues using a paper filter with microholes, (J. Prolab, São José dos Pinhais, PR, Brazil) and then sterilization (0.22 µm membrane filter).

#### 2.1.1. Determination of Soluble Solids Content

Three empty 25 mL beakers were weighed, and their respective weights were recorded. Subsequently, 5 mL of the rosemary hydroalcoholic extract were pipetted into each beaker and placed in a drying chamber at 80 °C for 24 h, until complete evaporation of the liquid. After drying, the beakers were placed in a desiccator until fully cooled and then weighed again. The amount of soluble solids content in the extract was calculated according to the following equation:

% soluble solids (m/v) = (m-b) × 100/Vo(1)

% soluble solids (m/m) = % soluble solid (m/v)/density(2)

where % m/V is the content of soluble solids; (m-b) is the mass of the beaker with the extract (m) minus the mass of the beaker without the extract (b); Vo is the volume of extract used for the analysis of soluble solids (5 mL).

#### 2.1.2. Evaluation of the Phytochemical Composition by High-Performance Liquid Chromatography (HPLC-DAD)

The identification of the compounds in the extract was performed by high-performance liquid chromatography (HPLC-DAD), from Merck-Hitachi (Darmstadt, Germany) model D-7000 with an automatic injector. The chromatographic conditions were as follows: mobile phase: formic acid (PA, Merck) diluted to 5% in ultrapure water (solvent A) and chromatographic methanol—Merck (solvent B). The flow rate was 1 mL/min, using a linear gradient. A chromatographic run of 45 min was conducted with a sample loading volume of 20 µL. Detection was carried out at wavelengths of 280 nm and 320 nm. A qualitative analysis of the compounds was performed and analyte identification was performed by using a spectral library.

#### 2.1.3. Evaluation of Phytochemical Composition by Gas Chromatography–Mass Spectrometry (GC-MS)

The analysis was performed using flow-modulated comprehensive two-dimensional gas chromatography, which comprised a TRACE 1300 gas chromatograph coupled to an ISQ mass spectrometer with a fast-scanning quadrupole mass analyzer (Thermo Scientific, Waltham, MA, USA). The equipment features a flame ionization detector (FID) and a split/splitless (SSL) injection system. Samples were introduced using an automatic TriPlus RSH sampler. Differential flow modulation in the ‘reverse fill/flush’ configuration was performed using the INSIGHT (SepSolve Analytical, Peterborough, UK) interface. The SSL injector and FID were maintained at 275 °C and 300 °C, respectively. The transfer line and ionization source were operated at 250 °C and 220 °C. The chromatographic oven was programmed to ramp from 60 °C to 240 °C at a rate of 3 °C min^−1^. Helium was used as the carrier gas (1.0 mL min^−1^) and as the modulating gas (7.5 mL min^−1^). The column set consisted of an HP-5 MS column (30 m × 0.25 mm id, df = 0.25 μm) and an HP-50 MS column (5 m × 0.25 mm id, df = 0.25 μm). The eluent from the secondary column was partitioned between the FID and MS in a ratio of 80% and 20%, respectively. The FID acquisition rate was 120 Hz. A scan range of 40–400 Th was employed to obtain the mass spectra. A solution of ethyl acetate at a concentration of 1.4 mg/mL was prepared for methylene essential oil, and 1.0 μL was injected into the chromatograph. Menthol was identified by matching its mass spectrum to the reference spectra in the NIST 2014 mass spectral library. For menthol quantification, a calibration curve was constructed using a commercial standard. A stock solution in ethyl acetate at a concentration of 0.8 mg/mL was prepared, and from this solution, concentrations of 0.4 mg/mL, 0.16 mg/mL, 0.08 mg/mL, and 0.04 mg/mL were prepared. Analyses were conducted under the conditions described above. Chromatogram processing was performed using GC Image (Zoex, Houston, TX, USA). Qualitative analysis and analyte identification were performed using mass spectral library searches with the NIST database and filtering using retention indices. A minimum MS match value of 800 was considered for putatively characterized compounds (level 3).

#### 2.1.4. Evaluation of the Free Radical Suppressive Activity (Antioxidant Activity)

This method is based on the reduction in the 2,2-Diphenyl-1-picrylhydrazyl radical (DPPH, Sigma-Aldrich, St Louis, MI, USA) in absolute ethyl alcohol solution, which presents a maximum absorption in the range 515–528 nm. The samples were prepared in different dilutions based on the total soluble solids (TSS) content, previously determined. The volume of extract necessary to prepare the dilutions at 1% *v*/*v* and 0.01% *v*/*v* was calculated. The calculated aliquot of the 1% *v*/*v* solution was added to a 10 mL flask, and the total volume was completed with ethanol. In 11 test tubes, numbered from 0 to 10, 1000, 960, 920, 880, 840, 800, 760, 720, 680, 640, or 600 µL of absolute alcohol were added, respectively, to each of them. Then, on top of the alcohol, 0, 40, 80, 120, 160, 200, 240, 280, 320, 360, or 400 mL of 0.01% extract were added. Tube 0 was the control (100% DPPH without the extract) and 1000 µL of DPPH was added into it. After 60 s, DPPH was added to the other tubes in 1 min intervals, following the numerical sequence of the tubes. After 30 min of adding DPPH to tube 0, the measurement was taken with a spectrophotometer (Micronal B582, São Paulo, Brazil) at 517 nm. The readings were made in triplicate, and the absorbance values were used to obtain the % of remaining DPPH versus the concentration of the plant extract (in μg/mL). Finally, the CE50 (concentration that eliminates 50% of free radicals) was calculated using the least squares method.

#### 2.1.5. Determination of Total Flavonoid Content

A stock solution was prepared by adding 100 µL of the extract to a 10 mL volumetric flask, followed by the addition of 9900 µL of methanol, resulting in a 1:99 ratio. This procedure was carried out in triplicate. Then, a 200 μL aliquot was taken from the stock solution and transferred into a 10 mL flask containing about 5 mL of methanol. Then, 200 μL of aluminum chloride (AlCl_3_) was added, and the volume was adjusted to approximately 5 mL of methanol. The mixture was stirred and incubated in a water bath at 20 °C for 30 min. Afterwards, the liquid level was adjusted, and the absorbance was recorded at 425 nm using a spectrophotometer (B582, Micronal, São Paulo, Brazil). The concentration of total flavonoids expressed as quercetin was calculated using the equation of the straight line from the standard curve.

#### 2.1.6. Determination of Total Phenol Content

A different stock solution was prepared. A 1 mL aliquot of the plant content was placed into a 100 mL volumetric flask, dissolved in a small amount of ethanol, and the volume was gradually brought up to the mark with distilled water while stirring (stock solution) (10 mg/mL). From this point, the procedure was performed in triplicate. A 200 µL aliquot was added to a 10 mL volumetric flask (1:50) containing about 5 mL of distilled water, followed by the addition of 800 µL of Folin–Ciocalteu reagent. The mixture was stirred for 5 s, and within 1 to 8 min, 1.2 mL of 20% sodium carbonate-tartrate buffer solution was introduced. The volume was then brought up to just below the meniscus with water, and the solution was incubated in a water bath at 20 °C. After 2 h, the meniscus was adjusted to the final volume at room temperature, stirred for 5 s, and absorbance was measured at 760 nm using a spectrophotometer. The concentration of total phenols expressed as gallic acid was calculated using the equation of the standard curve.

### 2.2. Strains

Two strains of each microorganism were utilized, including one clinical isolate and one standard ATCC strain, as detailed in Table 1. The clinical multidrug-resistant bacterial samples (*A.b.* CL, *P.a*. CL, *K.p.* CL) were provided by the clinical laboratories of the Policlin Saúde group and Valeclin Clinical Analysis Laboratory, both located in São José dos Campos, SP. The resistance profile for each strain was determined using the automated MicroSCAN AutoSCAN 4^®^ system. (Table 2, Table 3 and Table 4). The clinical sample of *C. albicans* was obtained from the Microbiology and Immunologyh Laboratory of the Dentistry Course at ICT—UNESP. The bacterial strains were initially isolated using Cetrimide agar (Kasvi, Brazil) for *P. aeruginosa* and McConkey agar (Kasvi, Brazil) for *A. baumannii* and *K. pneumoniae*, followed by cultivation (37 °C/24 h). Subsequently, they were inoculated for analysis, with the bacterial strains on BHI (Brain Heart Infusion—Kasvi, Brazil) agar and the fungal strains on Sabouraud-dextrose (SD—Kasvi, Brazil) agar, then incubated at 37 °C for 24 h. After incubation, microbial suspensions were prepared in which the colonies of the respective strains were diluted in sterile saline solution (NaCl 0.9%) and homogenized in a vortex mixer for 10 s for further standardization of the microbial solution according to the requirements of each protocol.

#### 2.2.1. Evaluation of the Antimicrobial Activity of the Hydroalcoholic Extract Against Planktonic Cultures

The microdilution method was performed following the Clinical and Laboratory Standards Institute (CLSI) guidelines, specifically documents M7-A9 (for MDR bacteria) and M27-A2 (for *C. albicans*), to determine the minimum inhibitory concentration (MIC) and minimum microbicidal concentration (MMC). Microbial suspensions were prepared in sterile saline solution (0.9% NaCl) with turbidity adjusted to 10^6^ colony-forming units per milliliter (CFU/mL), standardized using a spectrophotometer (Micronal, São Paulo, SP, Brazil). In different microplates (Kasvi K12-096, São José dos Pinhais, Brazil), 9 serial dilutions (1:2) of the extract were performed in Mueller Hinton (MH) culture medium (HiMedia^®^ Mumbai, India) for bacteria, and RPMI 1640 culture medium (with glutamine, without bicarbonate, and with phenol red indicator—INLAB) for *C. albicans*. The experimental groups were Negative control (C-), Sterility control (C.E), and Rosemary extract. Aliquots of 100 μL of each microbial suspension were added to all wells. The microplate was incubated at 37 °C for 24 h, and the MIC (Minimum Inhibitory Concentration) was identified as the well that exhibited no turbidity. To determine the MMC (Minimum Microbicidal Concentration), BHI agar was used for bacterial strains and SD agar for fungal strains, with an aliquot from each well of the microplate being inoculated using a sterile stick. After 48 h of incubation, colony growth was evaluated, and the MMC was determined as the lowest seeded concentration that showed no colony growth.

#### 2.2.2. Antibiofilm Evaluation of the Hydroalcoholic Extract

**Figure 1:**
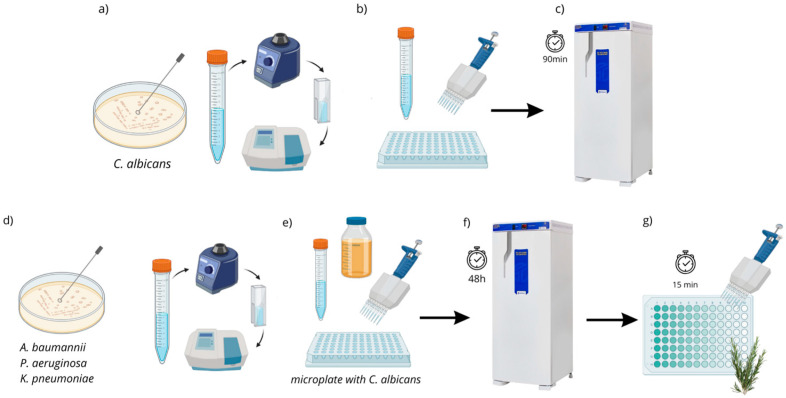
Step-by-step process for the formation of polymicrobial biofilm. Legend: (**a**–**c**) formation of the *C. albicans* biofilm; (**d**) standardization of each bacterial strain; (**e**,**f**) addition of culture medium and each bacterial strain to a microplate that already contains pre-formed *C. albicans* biofilm for incubation; (**g**) application of the plant extract. Source: Elaborated by the author.

To compose the polymicrobial biofilms, ATCC strains of *C. albicans* (18804) were combined with each of the ATCC strains of *A. baumannii* (19606), *P. aeruginosa* (15442), and *K. pneumoniae* (4352), while the clinical strain of *C. albicans* was combined with the respective clinical strains of the same multidrug-resistant microorganisms. Initially, standardization was performed using a spectrophotometer (B582, Micronal, São Paulo, Brazil) at 10^7^ CFU/mL to obtain *C. albicans*. Subsequently, 200 µL/well of the adjusted *Candida* suspension was added to microplates (Kasvi K12-096, São José dos Pinhais, Brazil) and incubated (37 °C/90 min) for the initial adhesion of fungal cells to the well (pre-adhesion period) [13]. Next, the supernatant was discarded, and 100 µL/well of BHI broth (Brain Heart Infusion—Kasvi, Brazil) was added. After the preparation and spectrophotometric standardization (10^7^ cells/mL) of the bacterial suspensions, 100 µL/well was added to the microplates, which already contained culture medium and fungal cells, resulting in a total volume of 200 µL/well. The plates were then incubated at 37 °C for 48 h to allow biofilm formation, with broth replacement after 24 h of incubation (Figure 1).

After biofilm formation, the biofilms were exposed for 15 min to the plant extract at different concentrations: 15 mg/mL, 7 mg/mL, 3.7 mg/mL, and 1.8 mg/mL. A culture medium served as the negative control, while 0.12% chlorhexidine gluconate, diluted 1:1 in culture medium, was used as the positive control. Each experimental group included n = 10 samples. After exposure to the treatments, the broth was discarded, and the wells were rinsed with sterile saline solution (0.9% NaCl) before performing cell viability analysis using the MTT assay (3-(4,5-Dimethylthiazol-2-yl)-2,5-Diphenyltetrazolium Bromide) (Sigma-Aldrich, Jurubatuba, Brazil). Then, 100 µL per well of the MTT solution (0.5 mg/mL) was added, and the plate was incubated in the dark at 37 °C for 1 h. After incubation, the MTT solution was removed and replaced with 100 µL of Dimethyl Sulfoxide (DMSO) (Synth, Diadema-SP, Brazil). The plate was then incubated again at 37 °C for 10 min and placed on a shaker under constant agitation for another 10 min. Subsequently, the optical densities (ODs) were measured using a microplate reader at 570 nm, and the obtained values were converted into a percentage of microorganism viability reduction using the formula below:

$$\%\;\mathrm{Viability}\;\mathrm{Reduction}=100-\left(\frac{OD\;treatment\;average}{OD\;negative\;control\;average}\times 100\right)$$

where

$$\mathrm{Average}=(\frac{sum\;of\;measurements}{10})$$

performed individually for the treatment groups, positive control and negative control.

### 2.3. Statistical Analysis

For the statistical evaluation of the in vitro experiments, normally distributed data were analyzed by ANOVA followed by Tukey’s test, whereas non-normally distributed data were assessed using the Kruskal–Wallis test and Dunn’s post hoc test. All analyses were conducted with GraphPad Prism 5.0, adopting a significance threshold of 5%.

## 3. Results

### 3.1. Phytochemical Analysis of the Extract

#### 3.1.1. Soluble Solids Content

The soluble solids content (TSS) indicates the amount of soluble substances in the hydroalcoholic extraction solvent (EtOH:H_2_O), thereby determining the final concentration of the plant extract. The quantification of the TSS for rosemary hydroalcoholic extract (RHE) obtained is presented in Table 5.

#### 3.1.2. Phytochemical Composition by HPLC

**Figure 2:**
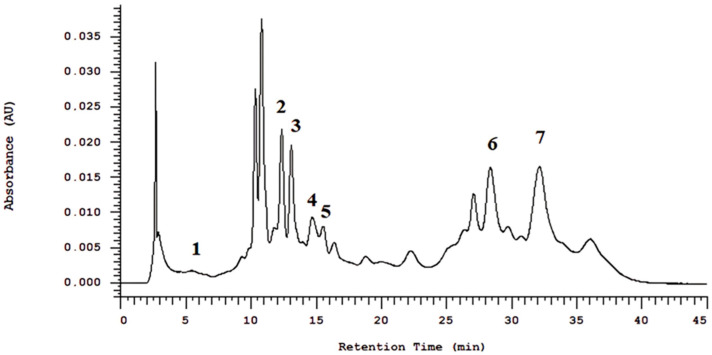
Chromatogram of rosemary hydroalcoholic extract (RHE). Legend: Identified compounds were numbered with their respective Retention Times (RT): (1) 5-O-caffeoylquinic acid—RT: 8.05 min; (2) 3,5-di-O-caffeoylquinic acid—RT: 12.35 min; (3) caffeoylquinic acid—RT: 13.20 min; (4) gallotannin—RT: 14.75 min; (5) gallotannin—RT: 15.55 min; (6) rosmarinic acid—RT: 28.50 min; (7) carnosic acid—RT: 32.05 min.

The HPLC chromatographic analysis showed peaks of 5-O-caffeoylquinic acid, 3,5-di-O-caffeoylquinic acid, caffeoylquinic acid, gallotannin, rosmarinic acid, and carnosic acid for RHE, as shown in Figure 2.

#### 3.1.3. Phytochemical Composition by GC-MS

**Figure 3:**
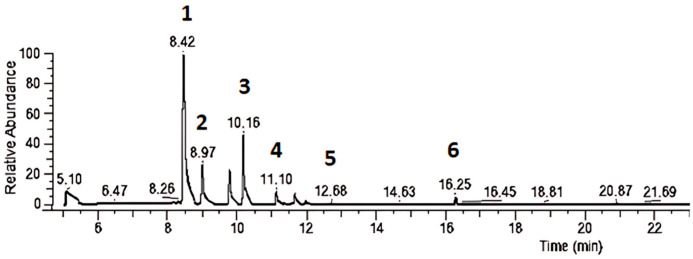
GC-MS Chromatogram of rosemary hydroalcoholic extract (RHE). Legend: Identified compounds were numbered with their respective Retention Times (RT): (1) α-Pinene—RT: 8.42 min; (2) 2,2-dimethyl-5-methylene norbornane—RT: 8.97 min; (3) β-Myrcene—RT: 10.15 min; (4) D-Limonene—RT: 11.10 min; (5) Cyclohexene, 1-methyl-4-(1-methylethylidene)—RT: 12.58 min; (6) Camphor—RT: 15.25 min.

The chromatographic analysis by gas chromatography (GC-MS) showed peaks of α-Pinene, 2,2-dimethyl-5-methylene norbornane, β-Myrcene, D-Limonene, Cyclohexene, 1-methyl-4-(1-methylethylidene), and Camphor for RHE, as shown in Figure 3.

#### 3.1.4. Free Radical Suppressing Activity of the Extract (Antioxidant Activity)

The antioxidant activity of the extract is shown in Table 6. The concentration that eliminates 50% of the free radicals (EC50) was 19.53 µg/mL.

#### 3.1.5. Quantification of Total Phenol and Flavonoid Content in the Extract

The extract was quantified and analyzed based on the construction of the standard curve for gallic acid, resulting in a total phenolic content of 7.70 mg/mL (0.77%). Using the quercetin standard curve, the total flavonoid content was analyzed, and the values are presented in Table 7. The flavonoid concentration was 5.12 mg/mL (0.512%).

### 3.2. Antimicrobial Activity in Planktonic Culture

#### Minimum Inhibitory Concentration (MIC) and Minimum Microbicidal Concentration (MMC)

The hydroalcoholic extract of rosemary (RHE) has an intense coloration, making it impossible to determine the Minimum Inhibitory Concentration (MIC) values due to the turbidity it caused, which prevented visual reading. The extract exhibited a Minimum Microbicidal Concentration (MMC) against all evaluated strains of *C. albicans*, *A. baumannii*, and *P. aeruginosa*. However, for *K. pneumoniae*, the MMC was only observed for the clinical strain (*K.p.* CL), as the ATCC strain was resistant to the tested concentrations. The MMC values are presented in Table 8.

### 3.3. Antibiofilm Activity of the Plant Extract

The concentrations used for biofilm analyses were selected based on the total soluble solids (TSS) content of the plant extract:

- 15 mg/mL (concentration equivalent to TSS—pure extract);
- 7.5 mg/mL (concentration equivalent to 1/2 of TSS);
- 3.7 mg/mL (concentration equivalent to 1/4 of TSS);
- 1.8 mg/mL (concentration equivalent to 1/8 of TSS).

Among the experimental groups, there were also the Control (−) (negative control—culture medium) and CHX (positive control—0.12% chlorhexidine digluconate).

#### 3.3.1. Action of Plant Extract on Polymicrobial Biofilm: *Candida albicans* + *Acinetobacter baumannii*

Regarding the biofilm composed of ATCC strains of *C. albicans* (18804) and *A. baumannii* (19606), a reduction in the percentage of viable microorganisms was observed in all groups treated with the plant extract for 15 min at different concentrations: 15 mg/mL (69.6%), 7.5 mg/mL (36.7%), 3.7 mg/mL (21.7%), and 1.8 mg/mL (19.4%). However, only the groups treated with the highest concentrations (15 and 7.5 mg/mL) showed a statistically significant difference (*p* < 0.05) when compared to the negative control group. Additionally, both were similar to the positive control, chlorhexidine gluconate (74.9%).

**Figure 4:**
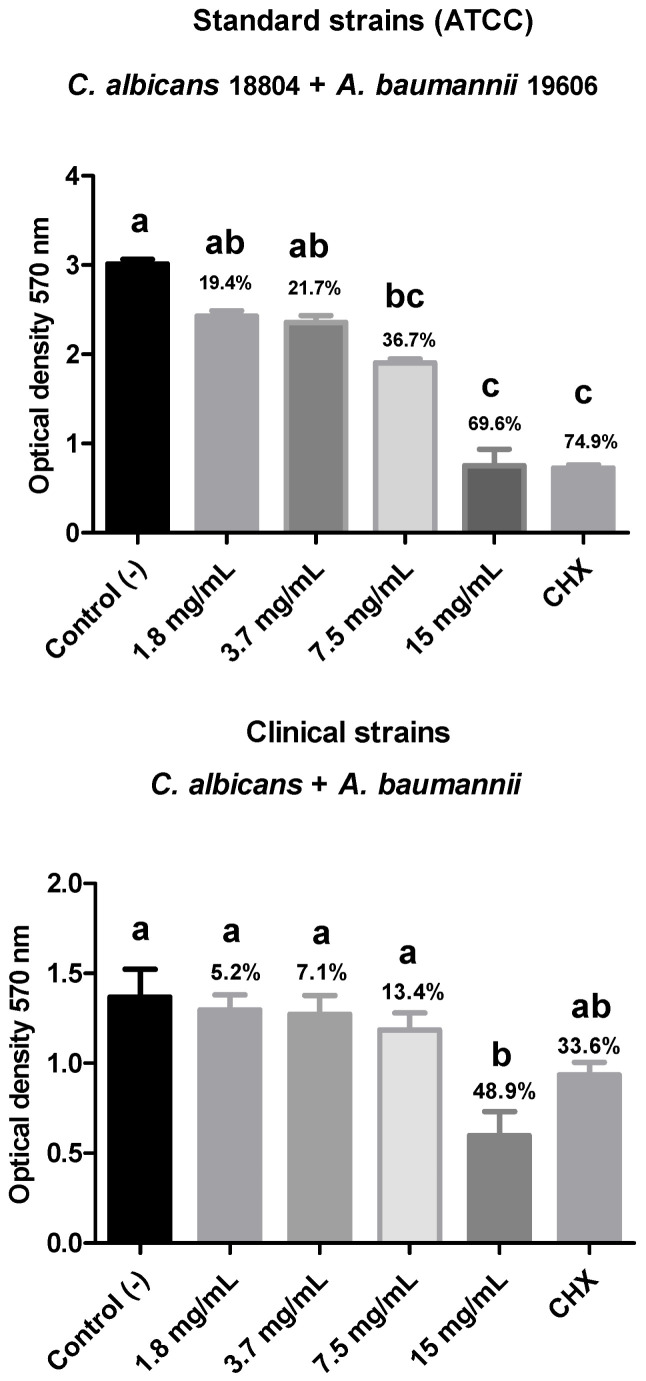
Evaluation of cell viability of the polymicrobial biofilm composed of the ATCC 18804 *C. albicans* + ATCC 19606 *A. baumanni* strains or clinical strains of *C. albicans* + multi-drug resistant *A. baumannii* after treatment with different concentrations of rosemary extract. Legend: Different letters indicate statistically different groups. Mean values ± standard deviation of the optical density (570 nm) reading of the polymicrobial biofilm of *C. albicans* and *A. baumannii* after 15 min of exposure to the concentrations of the plant extract, with C- (negative control—BHI broth), C+ (positive control—0.12% chlorhexidine digluconate). (n = 10. Kruskal–Wallis and Dunn’s test, *p* ≤ 0.05).

Regarding the biofilm composed of clinical strains of multidrug-resistant *A. baumannii* (*A.b.* CL) + *C. albicans* (*C.a.* CL), all treatment groups showed a reduction in the percentage of viable microorganisms in the polymicrobial biofilm. Notably, the group treated with 15 mg/mL exhibited a statistically significant difference (*p* < 0.05) compared to the negative control and a higher reduction percentage than the positive control (48.9% compared to the 33.6% biofilm reduction in the CHX-treated group) (Figure 4).

#### 3.3.2. Action of Plant Extract on Polymicrobial Biofilm: *Candida albicans* + *Pseudomonas aeruginosa*

**Figure 5:**
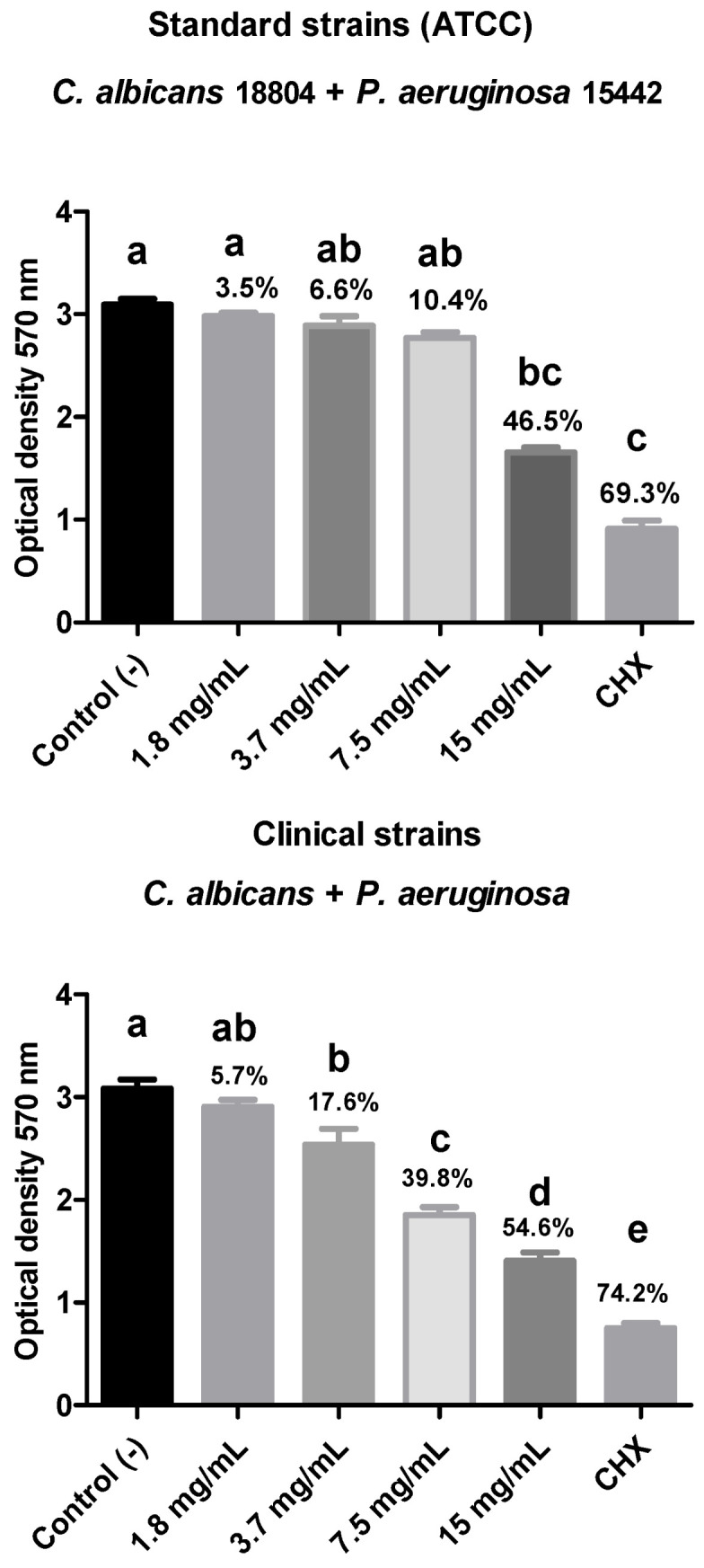
Evaluation of cell viability of the polymicrobial biofilm composed of the ATCC 18804 *C. albicans* + ATCC 15442 *P. aeruginosa* strains or clinical strains of *C. albicans* + multi-drug resistant *P. aeruginosa* after treatment with different concentrations of rosemary extract. Legend: Different letters indicate statistically different groups. Mean values ± standard deviation of the optical density (570 nm) reading of the polymicrobial biofilm of *C. albicans* and *P. aeruginosa* after 15 min of exposure to the plant extract concentrations, with C- (negative control—BHI broth) and C+ (positive control—0.12% chlorhexidine digluconate). (n = 10, One-way ANOVA with Tukey’s test, *p* ≤ 0.05).

Regarding the biofilm composed of the ATCC strains of *C. albicans* (18804) + *P. aeruginosa* (15442), there was a percentage reduction in viable microorganisms for all groups treated with the different dilutions of the plant extract, with the 15 mg/mL group standing out, reducing 46.5% of the polymicrobial biofilm, showing statistical equivalence with the CHX group (0.12% chlorhexidine digluconate) and a statistical difference (*p* < 0.05) with the negative control. The biofilm composed of the clinical strains of *C. albicans* + multi-drug-resistant *P. aeruginosa* also showed a reduction in the number of viable microorganisms in the tested treatment groups. Moreover, all treatment groups, except the 1.8 mg/mL group, showed a statistical difference with the negative control (*p* < 0.05) as well as between each other (Figure 5).

#### 3.3.3. Action of Plant Extract on Polymicrobial Biofilm: *Candida albicans* + *Klebsiella pneumoniae*

For the biofilm composed of ATCC strains of *C. albicans* (18804) + *K. pneumoniae* (4352), there was a percentage reduction in the number of viable microorganisms for all treatment groups. The 15 mg/mL group showed the highest reduction percentage (45.5%), with statistical equivalence to the positive control group (0.12% chlorhexidine digluconate), which reduced the biofilm by 48.2%. Both groups showed a statistically significant difference (*p* < 0.05) compared to the negative control.

**Figure 6:**
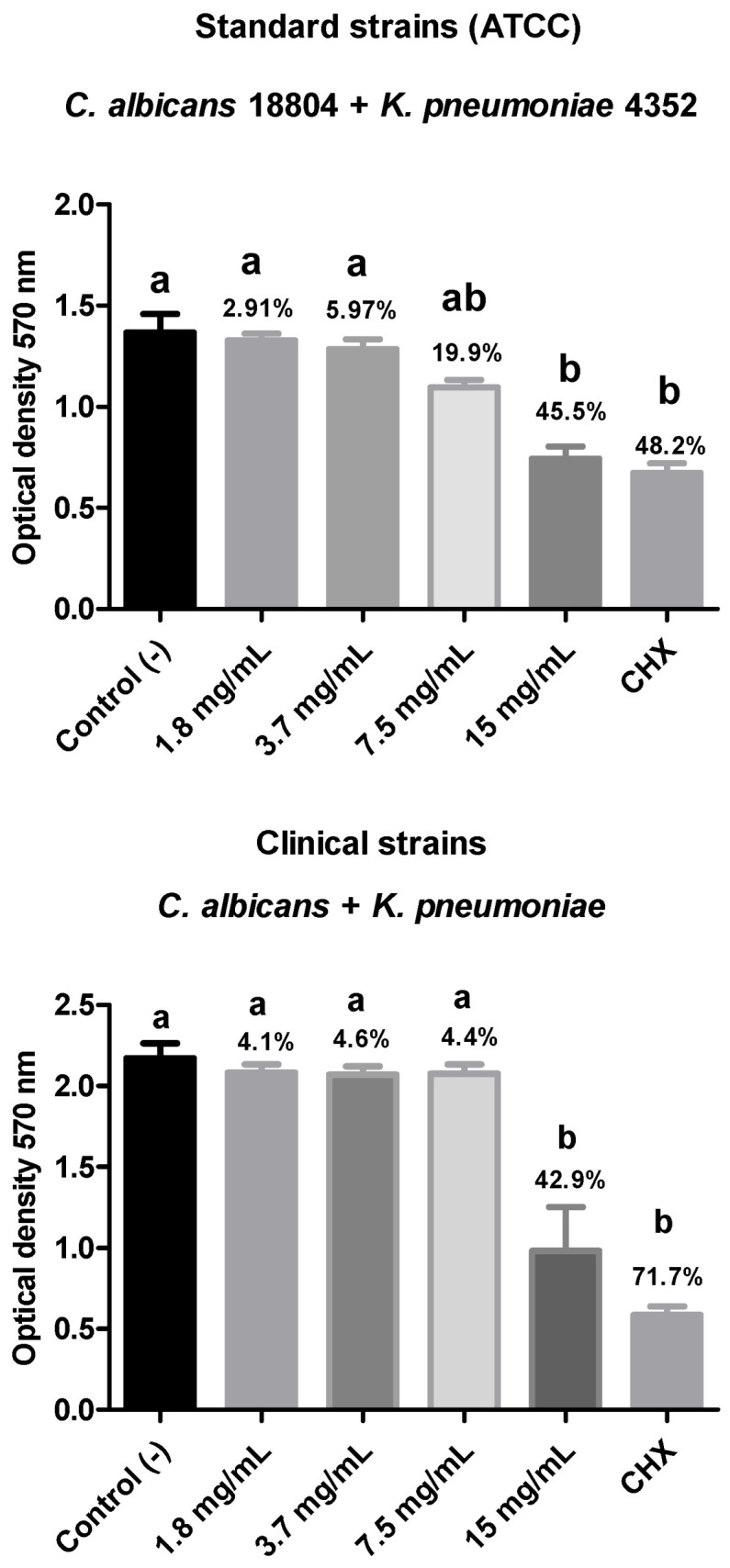
Evaluation of the cell viability of the polymicrobial biofilm composed of ATCC 18804 *C. albicans* + ATCC 4352 *K. pneumoniae* or clinical strains of *C. albicans* + multidrug-resistant *K. pneumoniae* after treatment with different concentrations of rosemary extract. Legend: Different letters indicate statistically different groups. Mean values ± standard deviation of the optical density (570 nm) reading of the polymicrobial biofilm of *C. albicans* and *K. pneumoniae* after 15 min of exposure to the plant extract concentrations, with C- (negative control—BHI broth) and C+ (positive control—0.12% chlorhexidine digluconate). (n = 10, Kruskal–Wallis and Dunn’s test, *p* ≤ 0.05).

For the biofilm composed of clinical strains of *C. albicans* + multidrug-resistant *K. pneumoniae*, there was a reduction in microorganism viability for all treatment groups. However, only the 15 mg/mL group showed a statistically significant difference from the negative control (*p* < 0.05), with a biofilm reduction percentage of 42.9%. This group was statistically similar to the positive control (CHX) (Figure 6).

## 4. Discussion

Given the significant challenge of finding new alternatives to combat antimicrobial-resistant pathogens, this study evaluated the phytochemical composition and potential of rosemary (*Rosmarinus officinalis* L.) extract in eliminating polymicrobial biofilms composed of *C. albicans* and *A. baumannii*; *C. albicans* and *K. pneumoniae*; and *C. albicans* and *P. aeruginosa*. The phytochemical profile of the plant extract was outlined, revealing antimicrobial effectiveness at different concentrations against planktonic cultures and polymicrobial biofilms of fungi and multidrug-resistant bacteria. As a result, the null hypothesis of this study was dismissed.

In 2017, the World Health Organization (WHO) released a priority list of pathogens for antimicrobial drug development, placing *A. baumannii* at the highest priority within the critical category [26]. By 2019, antimicrobial resistance was linked to an estimated 4.95 million deaths globally, with 1.27 million of those directly caused by bacterial infections from antibiotic-resistant pathogens [4].

Due to the growing global concern regarding multidrug-resistant bacteria, the medicinal properties of plants have gained attention as promising alternatives to conventional antibiotics, particularly in the form of phytotherapeutics [27,28].

The presence of phytochemical constituents in natural plant extracts—such as flavonoids, phenols, alkaloids, tannins, glycosides, saponins, and terpenoids—determines their diverse therapeutic properties [29]. Flavonoids and other phenolic compounds, for instance, are secondary metabolites found in various plant species and play crucial roles in their biological activities [30].

In this study, *R. officinalis* extract was found to contain flavonoids (5.12 mg/mL) and total phenols (7.70 mg/mL). A study by Megateli et al. (2018) [31] quantified phenolic compounds in *R. officinalis* using electromagnetic induction heating to enhance extraction. Under optimal conditions, the process yielded a maximum phenolic content of 127.87 ± 2.1 mg gallic acid equivalents per gram of dry weight and a total flavonoid content of 14.48 ± 1.5 mg quercetin equivalents per gram of dry weight.

Similarly, Bellumori et al. (2021) [32] conducted a phytochemical analysis using high-performance liquid chromatography (HPLC-DAD) and identified various phenolic compounds in *R. officinalis*, including rosmarinic acid, vanillic acid, cinnamic acid, rutin, kaempferol, trans-chalcone, and quercetin. In the present study, HPLC-DAD analysis of the hydroalcoholic extract of *R. officinalis* detected peaks corresponding to 5-O-caffeoylquinic acid, 3,5-di-O-caffeoylquinic acid, caffeoylquinic acid, gallotannin, rosmarinic acid, and carnosic acid.

Moreover, rosmarinic acid has the ability to integrate into the plasma membrane of microorganisms, compromising its structural integrity. This interaction leads to the leakage of ions, proteins, and other essential intracellular components, ultimately resulting in cell death. Additionally, contact between rosmarinic acid and microbial cells induces the generation of reactive oxygen species (ROS), which cause oxidative damage to critical biomolecules such as proteins, membrane lipids, and nucleic acids, thereby enhancing the compound’s antimicrobial effect [33,34,35].

For gas chromatography/mass spectrometry (GC/MS) analysis, the hydroalcoholic extract of *R. officinalis* in this study exhibited peaks for α-pinene, 2,2-dimethyl-5-methylene norbornane, β-myrcene, D-limonene, cyclohexene, 1-methyl-4-(1-methylethylidene), and camphor. In comparison, Naqvi et al. (2024) used reverse-phase high-performance liquid chromatography (RP-HPLC) to evaluate the active constituents of hydroethanolic *R. officinalis* extract, identifying diosmetin, rutin, and apigenin [36]. Hashemi et al. (2023) analyzed *R. officinalis* essential oil via GC/MS and found that the main components were α-pinene (24.6%), 1,8-cineole (14.1%), camphor (13.5%), camphene (8.1%), and limonene (6.1%) [37]. These variations across studies may be attributed to differences in extraction methods, solvent selection, and analytical technique parameters, which can influence the final composition of the extracts.

Antioxidants play a crucial role in preventing and treating diseases associated with oxidative damage, such as cancer, cardiovascular diseases, and neurodegenerative disorders [38,39]. Regarding the antioxidant activity of *R. officinalis*, the DPPH radical scavenging assay was employed, a widely used technique for assessing antioxidant capacity. The hydroalcoholic extract of *R. officinalis* exhibited an EC_50_ (effective concentration required to neutralize 50% of free radicals) of 19.53 µg/mL.

Another study evaluating *R. officinalis* essential oil reported an average yield of 0.92% and an antioxidant activity of 68.73 µg trolox g^−1^ ± 0.61, with a 63.5% reduction in DPPH radical levels. In a moisturizing fluid containing 0.5% *R. officinalis* essential oil, the antioxidant activity was measured at 37.3 µg trolox g^−1^ ± 8.21, with a 36% DPPH radical reduction [40]. Thomazini et al. (2021) also observed similar results, reporting an IC_50_ of 40.28 μg/mL for the aqueous extract of *R. officinalis* [41].

α-Pinene is a bicyclic monoterpene widely found in the essential oils of various plants and is one of the main constituents of rosemary (*Rosmarinus officinalis* L.) extract [42,43]. Studies have shown that α-pinene can modulate cellular pathways related to oxidative stress and inflammatory responses, highlighting its relevance in the phytotherapeutic use of rosemary [44]. α-Pinene exhibits significant antioxidant activity, attributed to its ability to scavenge free radicals and inhibit lipid peroxidation. This activity is associated with the presence of conjugated double bonds in its structure, which allow the stabilization of ROS. These mechanisms suggest that α-pinene may play a key role in cellular protection against oxidative damage, making it a promising compound for the development of natural antioxidant therapeutic agents.

Barros et al. (2023) evaluated its anti-biofilm activity against *Candida albicans*, and in vitro results indicated that the antifungal effect of α-pinene may be related to its interaction with ergosterol in the fungal cytoplasmic membrane. In the time-kill assay, the antifungal activity was not time-dependent and also inhibited biofilm formation, disrupting up to 88% of the existing biofilm [45].

In regard of microbiological analyses, the *R. officinalis* extract demonstrated bactericidal and fungicidal activity against all evaluated strains of *A. baumannii* (MMC of 1.88 mg/mL), *P. aeruginosa* (MMC of 1.88 mg/mL), and *C. albicans* (MMC 1.88 to 3.75 mg/mL). However, for *K. pneumoniae*, only the clinical strain (*K.p.* CL) exhibited inhibition (MMC of 3.75 mg/mL). The Minimum Inhibitory Concentration (MIC) values could not be determined due to extract-induced turbidity, which hindered visual reading. Therefore, MMC values were considered in this study.

Several studies have explored the antimicrobial potential of *R. officinalis* [13,21]. Kafa et al. (2022) investigated the antibacterial and antibiofilm activities of essential oils from *R. officinalis*, *Pelargonium graveolens*, and *Mentha piperita* against drug-resistant clinical strains of *A. baumannii*. The most potent antibacterial effects were observed with peppermint essential oil (2.5–5 μL/mL), followed by geranium (5–20 μL/mL) and rosemary (5–20 μL/mL) [46].

Another study evaluated the efficacy of *R. officinalis* essential oil against multidrug-resistant clinical bacterial pathogens. The results showed promising antibacterial activity against seven bacteria: *Escherichia coli*, *Enterobacter cloacae*, *Staphylococcus aureus*, *Serratia odorifera*, *K. pneumoniae*, *Klebsiella oxytoca*, and *Aeromonas hydrophila*, with MIC values of 35.7, 17.85, 71.4, 8.9, 17.8, 285.7, and 35.7 µg/mL, respectively, and MBC values of 142.8, 71.4, 285.7, 35.7, 71.4, 571.5, and 71.4 µg/mL, respectively. This study suggested that *R. officinalis* essential oil could be used as a therapeutic agent in combating a wide range of multidrug-resistant bacteria [47].

The antifungal effect of glycolic extract of *R. officinalis* was observed in the study by Meccatti et al. (2021) [13]. The minimum fungicidal concentration for *C. albicans, C. glabrata, C. krusei,* and *C. tropicalis* ranged from 25 to 50 mg/mL. Additionally, the authors assessed the antibiofilm potential of *R. officinalis* glycolic extract. Among the various forms of antimicrobial resistance, biofilm formation is one of the main resistance mechanisms. Biofilm is a structure composed of self-generated extracellular polymeric substances that encase bacteria and fungi on surfaces, with one of its functions being to protect them from environmental stress, preventing phagocytosis and promoting colonization [48]. The study cited earlier demonstrated that the glycolic extract of *R. officinalis* was effective in disrupting *C. albicans* biofilms after 30 min of exposure and *C. dubliniensis* biofilms after 24 h at a concentration of 50 mg/mL [13].

In the present study, the antibiofilm action of *R. officinalis* extract was evaluated against polymicrobial biofilms of *C. albicans* and multidrug-resistant bacteria (*C. albicans* + *A. baumannii*; *C. albicans* + *K. pneumoniae*; *C. albicans* + *P. aeruginosa*) after 15 min of contact using the MTT cell viability test. A reduction in biofilm viability was observed for all analyzed strains, with the polymicrobial biofilm composed of ATCC strains of *C. albicans* (18804) and *A. baumannii* (19606) showing the highest reduction in viable microorganisms (69.6%), demonstrating statistical equivalence to the positive control and statistical difference (*p* < 0.05) from the negative control.

According to the study by de Oliveira JR et al. (2017), *R. officinalis* extract was shown to be effective in reducing mono- and polymicrobial biofilms, effectively contributing to the in vitro control of significant microbial species responsible for various infections in the oral cavity and other body regions, such as *C. albicans*, *S. aureus*, *Enterococcus faecalis*, *Streptococcus mutans*, and *P. aeruginosa* [49]. Another study evaluated *R. officinalis* oil, which showed biofilm inhibition potential of *S. epidermidis* that was greater than 57% at a concentration of 25 μL ml^−1^, and a biofilm eradication of 67% was observed at a concentration of 50 μL ml^−1^ [50]. In another study on the in vivo prophylactic potential of *R. officinalis* glycolic extract, it was found that the extract is an excellent prophylactic antifungal agent at 6.25 mg/mL for 72 h and 12.5 mg/mL for 24 h in a *Galleria mellonella* larval model [51].

The reviewed literature revealed no studies addressing the action of *R. officinalis* extract against polymicrobial biofilms of the species combinations tested in this study. The present work presented important findings regarding the antimicrobial, antibiofilm, and chemical composition of the plant extract, revealing an exploitable potential for the formulation of new compounds. Future analyses may explore the development of a complementary antiseptic agent aimed at combating biofilms linked to hospital-acquired infections. Thus, this extract may be a promising therapeutic agent for controlling pathogenic microorganisms and biofilms.

## 5. Conclusions

In conclusion, biomolecules such as caffeoylquinic acid, rosmarinic acid, carnosic acid, α-Pinene, β-Myrcene, and D-Limonene were identified in the hydroalcoholic extract of *R. officinalis*. The plant extract exhibited compatible amounts of flavonoids and phenols, as well as antioxidant activity. Regarding antimicrobial action, the extract demonstrated microbicidal activity against bacterial and fungal strains and showed potential for the elimination of polymicrobial biofilms of *C. albicans* with multidrug-resistant bacteria, with biofilm reduction percentages of up to 69.6% (*p* < 0.05) and superior performance compared to 0.12% chlorhexidine against *C. albicans* + *A. baumannii* biofilm.

## Figures and Tables

**Table 1:** Strains used in the experiments.

Strains	ATCC	Clinic
*C. albicans*	18804	*C.a.* CL
*A. baumannii*	19606	*A.b.* CL
*P. aeruginosa*	15442	*P.a.* CL
*K. pneumoniae*	4352	*K.p.* CL

**Table 2:** Antibiotic resistance profile of *A. baumannii* clinical strain.

Antibiotics	*A. baumannii* Clinical Strain (*A.b.* CL)
Ertapenem	Resistant
Imipenem	Resistant
Meropenem	Resistant

Source: Data provided by the Bioclin Laboratory of the Policlin group, São José dos Campos—SP, Brazil.

**Table 3:** Antibiotic resistance profile of *P. aeruginosa* clinical strain.

Antibiotics	*P. aeruginosa* Clinical Strain (*P.a.* CL)
Amikacin	Sensible
Cefepime	Intermediary
Ceftazidime	Intermediary
Ciprofloxacin	Intermediary
Imipenem	Intermediary
Levofloxacin	Intermediary
Meropenem	Sensible
Piperacillin—Tazobactam	Intermediary

Source: Data provided by the Bioclin Laboratory of the Policlin group, São José dos Campos—SP, Brazil.

**Table 4:** Antibiotic resistance profile of *K. pneumoniae* clinical strain.

Antibiotics	*K. pneumoniae* Clinical Strain (*K.p.* CL)
Amikacin	Sensible
Cefepime	Intermediary
Ceftazidime	Intermediary
Ciprofloxacin	Intermediary
Imipenem	Intermediary
Levofloxacin	Intermediary
Meropenem	Sensible
Piperacillin—Tazobactam	Intermediary

Source: Data provided by the Bioclin Laboratory of the Policlin group, São José dos Campos—SP, Brazil.

**Table 5:** Soluble solids content of rosemary hydroalcoholic extract (RHE).

Extract	Extraction Type	TSS (mg/mL)
Rosemary hydroalcoholic extract (RHE)	EtOH:H_2_O (50:50)	15.50 ± 0.06

**Table 6:** Antioxidant activity of rosemary hydroalcoholic extract (RHE).

Extract	Extraction Type	C_E_50 (µg/mL)
Rosemary hydroalcoholic extract (RHE)	EtOH:H_2_O (50:50)	19.53 ± 5.43

Legend: EtOH—absolute ethyl alcohol; H_2_O—distilled water; C_E_50—concentration that eliminates 50% of free radicals.

**Table 7:** Total phenol and flavonoid content of rosemary hydroalcoholic extract (RHE).

Extract	Extraction Type	FNS (mg/mL)	FLV (mg/mL)
Rosemary hydroalcoholic extract (RHE)	EtOH:H_2_O (50:50)	7.70 ± 0.08	5.12 ± 0.09

Legend: EtOH—absolute ethyl alcohol; H_2_O—distilled water; FNS—Total phenols; FLV—Total flavonoids.

**Table 8:** Minimum Microbicidal Concentration of rosemary hydroalcoholic extract (RHE).

	Strains	RHE
		CMM (mg/mL)
*A. baumannii*	19606	1.88
	*A.b.* CL	1.88
*P. aeruginosa*	15442	1.88
	*P.a.* CL	1.88
*K. pneumoniae*	4352	-
	*K.p.* CL	3.75
*C. albicans*	18804	3.75
	*C.a.* CL	1.88

Legend: MMC—Minimum Microbicidal Concentration; RHE—rosemary hydroalcoholic extract; CL—clinical strain.

## Data Availability

The original contributions presented in this study are included in the article. Further inquiries can be directed to the corresponding author.
